# The Association between Platelet Glycocalicin and High Microsatellite Instability in Colorectal Cancer

**DOI:** 10.1155/2022/9012063

**Published:** 2022-04-07

**Authors:** Zeng-yao Liu, Qing-chun Jia, Wen Wang, Yu-xi Liu, Rui-tao Wang, Jia-yu Li

**Affiliations:** ^1^Department of Internal Medicine, Harbin Medical University Cancer Hospital, Harbin Medical University, Harbin, Heilongjiang 150081, China; ^2^Department of Interventional Medicine, The First Affiliated Hospital, Harbin Medical University, Harbin, Heilongjiang 150001, China; ^3^Institute of Intensive Care Medicine, Heilongjiang Academy of Medical Science, Harbin, Heilongjiang 150081, China

## Abstract

**Background:**

Elevated platelet volume is the risk factor for the development and poor overall survival of colorectal cancer (CRC) patients. Both microsatellite status and platelet glycoprotein Ib*α* (GPIb*α*) are related to platelet volume in CRC patients. This study aimed to investigate platelet GPIb*α* ectodomain (termed glycocalicin) levels among CRC patients and the association between the glycocalicin levels and microsatellite status in CRC.

**Methods:**

The clinical and laboratory data of 430 CRC patients between January 2018 and December 2018 in Harbin Medical University Cancer Hospital were collected. The microsatellite status was determined with a polymerase chain reaction. The participants were separated into high microsatellite instability (MSI-H) and microsatellite stable (MSS) groups according to microsatellite status. The glycocalicin levels were measured with an enzyme-linked immunosorbent assay, and the cut-off point was determined with the receiver-operating characteristics curve. The clinical and pathological characteristics were collected via electronic medical records. Logistic regression was used to explore the association between glycocalicin and microsatellite status.

**Results:**

Among the 430 CRC patients enrolled, 64 patients (14.9%) were identified as MSI-H and others as MSS CRC. Glycocalicin levels were significantly reduced in patients with MSI-H than those with MSS. After controlling for potential confounders, logistic regression analysis revealed that glycocalicin levels were independently associated with MSI-H CRC.

**Conclusions:**

Reduced glycocalicin levels are associated with the MSI-H subtype of CRC. Further research is needed to elucidate the mechanisms of the association between glycocalicin and MSI-H in CRC patients.

## 1. Introduction

The occurrence of colorectal cancer involves the gradual accumulation and malignant transformation of various genetic changes, which are conducive to the proliferation and growth of tumor cells [1]. Microsatellite instability (MSI) results from DNA mismatch repair system defects, characterized by a significant increase in the intragenic mutation rate of short tandem repeat DNA sequences called microsatellites [2]. CRC tumors can be classified into high MSI (MSI-H), low MSI (MSI-L), and microsatellite stable (MSS) according to their mutation patterns and the proportion of markers showing MSI [1]. Approximately 15% of CRC patients demonstrate MSI-H tumors [3]. Patients with MSI-H CRC generally have a better prognosis and a more effective immune response than patients with MSS CRC [4]. MSI-H CRC patients usually have lower platelet volume [4, 5].

Glycoprotein (GP) Ib is a complex formed by the linking of GPIb*α* with two GPIb*β* subunits through a membrane-proximal disulfide bond. The number of GPIb on the platelet surface is strongly associated with platelet volume [6]. GPIb receptors are more abundant in larger platelets [5]. As a specific platelet adhesion receptor, GPIb*α* is an essential platelet function regulator, regulating the survival and clearance of platelets [7, 8]. The amount of GPIb*α* could induce signals to enter platelets across the membrane by binding to the Willebrand factor (VWF), leading to platelet activation and subsequent platelet aggregation and thrombosis [9]. GPIb*α* releases glycocalicin (extracellular domain of GPIb*α*) into the plasma after binding with VWF [10–13].

Both MSI-H subtype and amount of GPIb are related to the platelet size. The levels of glycocalicin depend on the levels of GPIb*α*. However, there is limited evidence to show the association between glycocalicin and microsatellite status among CRC patients. This study aimed to examine glycocalicin levels in CRC patients and investigate the association between glycocalicin levels and MSI status.

## 2. Material and Methods

### 2.1. Study Population

This study reviewed 430 CRC patients at the Harbin Medical University Cancer Hospital from January 2018 to December 2018 via an online medical system. CRC was confirmed with a pathological diagnosis. Patients were excluded under the following conditions: (1) with infection, autoimmune disease, hematological disorders, hypertension, and diabetes mellitus; (2) with a history of cancer other than CRC; (3) with a history of chemotherapy or radiotherapy or medical treatment with acetylic salicylic acid; and (4) with insufficient information.

### 2.2. Clinical Examination and Biochemical Measurements

Clinicopathological information was collected via the medical records: age, gender, body mass index (BMI), carcinoembryonic antigen (CEA), smoking status, drinking status, albumin, creatinine, white blood cells (WBC), hemoglobin, platelet count, tumor location, tumor size, histological type, histological grade, venous invasion, perineural invasion, T classification, lymph node metastasis, and distant metastasis. Two pathologists independently evaluated the pathological tumor stage based on the 8th edition of the American Joint Committee on Cancer (AJCC) cancer staging system. Another pathological expert in CRC was invited to decide if the diagnosis was inconsistent between these two pathologists. Venous blood samples were collected from all participants with sodium citrate tubes under fasting conditions before any treatment. Each blood sample was centrifuged at 2,500 rpm for 10 minutes, and the supernatant was kept frozen at -80°C until assayed. Routine blood tests were conducted in the hospital's clinical laboratory. Glycocalicin was measured with an enzyme-linked immunosorbent assay (ELISA) kit (*CUSABIO*, Wuhan, China) based on the manufacturer's instructions. Each sample was tested in duplicate. The intra- and inter-assay variations were below 8%.

### 2.3. MSI Analysis

MSI was evaluated with DNA obtained from freshly frozen tumor tissue samples, using polymerase chain reaction and amplified microsatellite-labeled primers, including BAT25, BAT26, NR-21, NR-24, and NR-27. The MSI-H was defined as at least three of the five markers being unstable, or it was described as MSS when the unstable markers were fewer than three. No sample in this study had only two unstable markers.

### 2.4. Statistical Analysis

The data were presented as the mean ± standard deviation (SD) for normally distributed data, the median (interquartile range) for not normally distributed data, and frequencies for categorical data. Normally distributed continuous variables in the two groups were compared with Student's *t* test and skewed-distributed with the Mann–Whitney *U* test. The chi-square test was used for categorical variables. Binary logistic regression was used to assess clinicopathological factors associated with MSI-H status, reporting an adjusted odds ratio with 95% confidence interval (AOR, 95% CI). The statistical analyses were conducted using SPSS Statistics version 25.0 (SPSS Inc., Chicago, IL, USA). Receiver-operating characteristics (ROC) curve analysis was performed to calculate the area under the curve (AUC) and evaluate the optimal cut-off point of glycocalicin using MedCalc version 15.0. Differences were considered significant when *p* < 0.05.

## 3. Results

**Figure 1:**
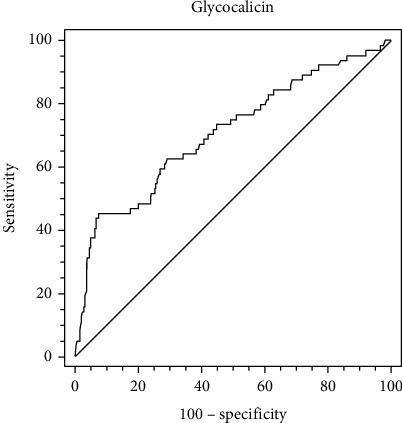
Optimal cut-off value was determined for glycocalicin using standard receiver-operating characteristic curve analysis.

The median value of glycocalicin among all patients was 26.5 (range, 11.7-51.5). According to the MSI-H, the optimal cut-off value of glycocalicin was determined with ROC analysis (Figure 1). Specificity was 45.3% and sensitivity was 92.6% (AUC =0.708, 95% CI: 0.632-0.783, *p* 0.001). According to the cut-off value (20.5 ng/mL), there were 374 patients (87.0%) in the group with glycocalicin levels >20.5 ng/mL and 56 patients (13.0%) in the group with glycocalicin levels ≤20.5 ng/mL. Baseline characteristics were compared between the two groups, and only the MSI status was significantly different with *p* < 0.001 (Table 1).

**Figure 2:**
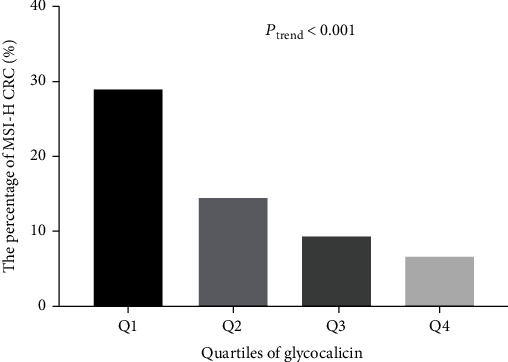
The association between high microsatellite instability and glycocalicin levels among colorectal cancer patients.

All CRC patients were classified into quartiles according to their glycocalicin levels, including Q1 ≤ 23.2 ng/mL, 23.2 ng/mL < Q2 ≤ 26.3 ng/mL, 26.3 ng/mL < Q3 ≤ 29.6 ng/mL, and Q4 >29.6 ng/mL (Figure 2). The percentages of patients with MSI-H in each group were 29.0%, 14.5%, 9.4%, and 6.5%, respectively. The results showed that elevated glycocalicin levels were associated with MSI-H (*p* < 0.001).

The baseline characteristics of 430 participants were compared between the MSI-H and MSS groups in Table 2. Patients in the MSI-H group were younger (*p* < 0.05) and had higher BMI (*p* < 0.005) and levels of WBC (*p* < 0.005) than those in the MSS group. Lower levels of glycocalicin levels (*p* < 0.001), hemoglobin (*p* < 0.05), and CEA (*p* < 0.05) were found in the MSI-H group. Tumor characteristics, including tumor location, tumor size, lymphatic invasion, lymph node metastasis, clinical stage, and histological type, were significantly different between the MSI-H and MSS groups (*p* < 0.05). The final logistic regression model included all significant variables in the univariate analysis. Patients with a higher BMI, higher WBC levels, tumor size 5.0 cm, proximal CRC, and mucinous type were more likely to have MSI-H. Notably, decreased glycocalicin levels were associated with MSI-H after adjusting for other confounding variables (AOR, 0.854; 95% CI, 0.801-0.910; *p* < 0.001) (Table 3).

## 4. Discussion

Glycocalicin levels were significantly reduced in MSI-H CRC patients compared with MSS CRC patients. Glycocalicin levels were strongly associated with MSI-H status in CRC patients after controlling for potential confounders.

MSI status is a critical factor in the pathogenesis of CRC. It could also be used as an essential molecular marker for the prognosis and adjuvant therapy of CRC [14, 15]. MSI-H tumors are more likely to be mucinous and poorly differentiated [1]. Regardless of the depth of tumor invasion, CRC with the MSI-H phenotype is unlikely to spread to regional lymph nodes or distant organs [16, 17]. MSI-H CRC responds well to immunotherapy [18]. Age under 50 is a strong predictor of MSI, and younger patients are more likely to have MSI-H CRC [19]. In our study, patients in the MSI-H group had a younger age than those in the MSS group. In our study, patients with proximal CRC and mucinous type CRC tend to be MSI-H type, which also consists of the current evidence. MSI-H is more common in proximal colon cancer than in distal colon cancer [20, 21].

The exact mechanisms of decreased glycocalicin in MSI-H CRC are still unclear. Mean platelet volume (MPV) refers to platelet average size and indicates platelet activation [4, 5]. Activated platelets can cause tumor proliferation, angiogenesis, and increase tumor adhesion, promoting tumor development and metastasis [22]. Lower MPV levels are independently associated with better overall survival of CRC [23]. Moreover, the decreased MPV levels are associated with MSI-H CRC, and MSI-H CRC patients exhibited a better prognosis than MSI-L CRC patients [4, 24]. The evidence about the role of GPIb or glycocalicin levels in the occurrence or prognosis of CRC is limited. GPIb receptors are expressed in lower numbers in smaller platelets [5, 6]. As mentioned before, glycocalicin is the extracellular domain released by GPIb*α*. Hence, glycocalicin levels fluctuate based on GPIb*α* levels. This consistent evidence suggests that platelet volume might play a role in the association between MSI-H and glycocalicin in CRC patients.

The elevated GPIb levels are related to the tendency to thrombosis [6]. Blocking the ligand-binding area of GPIb*α* can effectively inhibit tumor metastasis and improve the carcinogenic environment in cancer patients [25, 26]. The shedding of glycocalicin occurs when GPIb*α* binds to VWF, followed by the increased glycocalicin content in plasma [7]. Increased VWF is a risk factor for venous thromboembolism and long-term venous complications [27]. Microsatellite has been reported as a part of the VWF promoter region and promotes glucocorticoid-induced VWF levels [28]. Thus, VWF might be associated with our current findings. However, further study is required to investigate the association between MSI status and VWF in CRC patients.

Several limitations in this study should be considered. Our results were sourced from a single hospital. Only Chinese participants were included, so the results need to be confirmed in other ethnic populations. Further study with a potential mechanistic explanation is required.

## 5. Conclusions

Reduced glycocalicin is associated with the MSI-H subtype of CRC. Further mechanistic research is needed to explain the findings, which might help guide management strategies in CRC patients.

## Figures and Tables

**Table 1:** Baseline characteristics of CRC patients according to glycocalicin levels.

Variables	Total	Glycocalicin ≤20.5 ng/mL	Glycocalicin >20.5 ng/mL	*p* value
Age (years)				0.575
≤65	309 (71.9)	42 (75.0)	267 (71.4)	
>65	121 (28.1)	14 (25.0)	107 (28.6)	
Gender (%)				0.581
Male	245 (57.0)	30 (53.6)	215 (57.5)	
Female	185 (43.0)	26 (46.4)	159 (42.5)	
BMI (kg/m^2^)	23.4 ± 3.2	23.9 ± 3.6	23.3 ± 3.2	0.199
Current smoker (%)				0.809
Yes	183 (42.6)	23 (41.1)	160 (42.8)	
No	247 (57.4)	33 (58.9)	214 (57.2)	
Drinker (%)				0.375
Yes	139 (32.3)	21 (37.5)	118 (31.6)	
No	291 (67.7)	35 (62.5)	256 (68.4)	
WBC (×10^9^/L)	7.09 ± 2.44	7.23 ± 2.43	7.07 ± 2.45	0.657
Hemoglobin (g/L)	132.9 ± 23.4	128.0 ± 27.0	133.6 ± 22.8	0.138
Platelet count (×10^9^/L)	269.7 ± 87.8	278.5 ± 96.1	268.3 ± 86.6	0.417
Creatinine (*μ*mol/L)	81.1 ± 18.6	80.4 ± 12.5	81.2 ± 19.3	0.790
CEA (ng/mL)	4.37 (2.03-11.31)	3.49 (1.86-12.04)	4.52 (2.12-11.07)	0.499
Tumor size (cm)				0.208
<5.0	278 (64.7)	32 (57.1)	246 (65.8)	
≥5.0	152 (35.3)	24 (42.9)	128 (34.2)	
Tumor location (%)				0.051
Proximal	157 (36.5)	27 (48.2)	130 (34.8)	
Distal	273 (63.5)	29 (51.8)	244 (65.2)	
Histological type (%)				0.756
Non-mucinous	307 (71.4)	39 (69.6)	268-71.7	
Mucinous	123 (28.6)	17 (30.4)	106 (28.3)	
Histological grade (%)				0.147
Well/moderately differentiated	359 (83.5)	43 (76.8)	316 (84.5)	
Poorly differentiated	71 (16.5)	13 (23.2)	58 (15.5)	
Lymphatic invasion (%)				0.185
Absent	331 (77.0)	47 (83.9)	284 (75.9)	
Present	99 (23.0)	9 (16.1)	90 (24.1)	
Perineural invasion (%)				0.545
Absent	365 (84.9)	48 (85.7)	317 (84.8)	
Present	65 (15.1)	8 (14.3)	57 (15.2)	
T classification (%)				0.372
T1+T2	63 (14.7)	6 (10.7)	57 (15.2)	
T3+T4	367 (85.3)	50 (89.3)	317 (84.8)	
Lymph node metastasis (%)				0.511
Absence	267 (62.1)	37 (66.1)	230 (61.5)	
Presence	163 (37.9)	19 (33.9)	144 (38.5)	
Distant metastasis (%)				0.426
Absence	382 (88.8)	48 (85.7)	334 (89.3)	
Presence	48 (11.2)	8 (14.3)	40 (10.7)	
Stage (%)				0.682
I-II	258 (60.0)	35 (62.5)	223 (59.6)	
III-IV	172 (40.0)	21 (37.5)	151 (40.4)	
MSI status (%)				< 0.001
MSS	366 (85.1)	27 (48.2)	339 (90.6)	
MSI-H	64 (14.9)	29 (51.8)	35 (9.4)	

Data are presented as means (standard deviation), median (interquartile range), or number (percentage). BMI: body mass index; WBC: white blood cells; CEA: carcinoembryonic antigen; MSS: microsatellite stable; MSI: microsatellite instability; MSI-H: high MSI.

**Table 2:** Clinicopathological characteristics of the CRC patients according to microsatellite instability status.

Variables	Total	MSI-H	MSS	*p* value
Number	430	64	366	
Age (years)	59.4 ± 10.0	56.4 ± 11.8	59.9 ± 9.6	0.029
Gender (female, %)	181 (42.1)	33 (51.6)	148 (40.4)	0.096
BMI (kg/m^2^)	23.4 ± 3.2	24.5 ± 3.3	23.2 ± 3.2	0.004
Current smoker (%)	183 (42.6)	25 (39.1)	158 (43.2)	0.540
Drinker (n, %)	139 (32.3)	17 (26.6)	122 (33.3)	0.285
Creatinine (*μ*mol/L)	81.1 ± 18.6	80.9 ± 19.7	81.1 ± 18.4	0.929
CEA (ng/mL)	3.14 (1.57-7.98)	3.14 (1.57-7.98)	4.86 (2.15-12.12)	0.030
WBC (×10^9^/L)	7.09 ± 2.44	8.07 ± 2.98	6.92 ± 2.30	0.004
Hemoglobin (g/L)	132.9 ± 23.4	124.6 ± 27.3	134.3 ± 22.4	0.009
Platelet count (×10^9^/L)	269.7 ± 87.8	286.2 ± 112.6	266.8 ± 82.6	0.103
Glycocalicin (ng/mL)	26.5 ± 6.0	22.6 ± 6.3	27.2 ± 5.6	< 0.001
Tumor location (%)				< 0.001
Proximal	157 (36.5)	38 (59.4)	119 (32.5)	
Distal	273 (63.5)	26 (40.6)	247 (67.5)	
Tumor size (cm, %)				0.011
<5.0	282 (65.6)	33 (51.6)	249 (68.0)	
≥5.0	148 (34.4)	31 (48.4)	117 (32.0)	
Histological grade (%)				0.419
Well/moderately differentiated	307 (71.4)	43(67.2)	264 (72.1)	
Poorly differentiated	123 (28.6)	21 (32.8)	102 (27.9)	
Histological type (%)				0.007
Non-mucinous	359 (83.5)	46 (71.9)	313 (85.5)	
Mucinous	71 (16.5)	18 (28.1)	53 (14.5)	
Lymphatic invasion (%)				0.030
Absent	331 (77.0)	56 (87.5)	275 (75.1)	
Present	99 (23.0)	8 (12.5)	91 (24.9)	
Perineural invasion (%)				0.527
Absent	365 (84.9)	56 (87.5)	309 (84.4)	
Present	65 (15.1)	8 (12.5)	57 (15.6)	
T classification (%)				0.811
T1+T2	63 (14.7)	10 (15.6)	53 (14.5)	
T3+T4	367 (85.3)	54 (84.4)	313 (85.5)	
Lymph node metastasis (%)				0.010
Absence	267 (62.1)	49 (76.6)	218 (59.6)	
Presence	163 (37.9)	15 (23.4)	148 (40.4)	
Distant metastasis (%)				0.713
Absence	382 (88.8)	56 (87.5)	326 (89.1)	
Presence	48 (11.2)	8 (12.5)	40 (10.9)	
Stage (%)				0.003
I-II	258 (60.0)	49 (76.6)	209 (57.1)	
III-IV	172 (40.0)	15 (23.4)	157 (42.9)	

Data are presented as means (standard deviation), median (interquartile range), or number (percentage). Abbreviations see Table 1.

**Table 3:** Logistic regression analysis for the associations between glycocalicin and microsatellite instability status among colorectal cancer patients.

Variables	*β*	AOR (95% CI)	*p* value
Age (years)	-0.018	0.982 (0.949-1.015)	0.280
BMI (kg/m^2^)	0.117	1.124 (1.020-1.239)	0.018
WBC (×10^9^/L)	0.178	1.195 (1.055-1.353)	0.005
Hemoglobin (g/L)	-0.012	0.988 (0.975-1.001)	0.063
CEA (ng/ml)	-0.004	0.996 (0.985-1.007)	0.471
Glycocalicin (ng/mL)	-0.158	0.854 (0.801-0.910)	< 0.001
Tumor size (cm) (≥ 5.0 vs <5.0)	1. 000	2.718 (1.411-5.234)	0.003
Tumor location (proximal vs distal)	1.146	3.146 (1.646-6.014)	0.001
Histological type (mucinous vs non-mucinous)	0.795	2.214 (1.040-4.713)	0.039
Lymphatic invasion (presence vs absence)	-0.460	0.631 (0.230-1.729)	0.371
Lymph node metastasis (presence vs absence)	-0.020	0.980 (0.145-6.649)	0.984
Stage (III+IV vs I+II)	-0.901	0.406 (0.061-2.727)	0.354

All significant variables in univariate analysis were included in the final logistic regression model. Data are presented as coefficient *β*, and adjusted odds ratio (AOR) with 95% confidence interval (CI). Abbreviations see Table 1.

## Data Availability

The datasets generated and analyzed during the current study are not publicly available due to data protection regulations concerning patient information, but are available from the corresponding author upon reasonable request.

## Abbreviations

MPV: Mean platelet volume
CRC: Colorectal cancer
GPIb*α*: Glycoprotein Ib*α*
MSI: Microsatellite instability
MSI-H: High MSI
MSI-L: Low MSI
MSS: Microsatellite stable
VWF: Willebrand factor
BMI: Body mass index
CEA: Carcinoembryonic antigen
WBC: White blood cells
AJCC: American Joint Committee on Cancer
SD: Standard deviation
AOR: Adjusted odds ratio
CI: Confidence interval
AUC: Area under the curve.
